# Malaria-visceral leishmaniasis co-infection and associated factors among migrant laborers in West Armachiho district, North West Ethiopia: community based cross-sectional study

**DOI:** 10.1186/s12879-019-3865-y

**Published:** 2019-03-08

**Authors:** Yibeltal Aschale, Animen Ayehu, Ligabaw Worku, Habtie Tesfa, Meseret Birhanie, Wossenseged Lemma

**Affiliations:** 1 Department of Medical Parasitology, College of Health Sciences, Debre Markos University, P.O.Box: 269, Debre Markos, Ethiopia; 2 Department of Medical Parasitology, College of Medicine and Health Sciences, Bahir Dar University, Bahir Dar, Ethiopia; 3 Department of Medical Parasitology, School of Biomedical and Laboratory Sciences, College of Medicine and Health Sciences, University of Gondar, Gondar, Ethiopia

**Keywords:** Visceral leishmaniasis, Malaria, Co-infection, Migrant laborers

## Abstract

**Background:**

Malaria and leishmaniasis are the two largest parasitic killers in the world. Due togeographical overlap of these diseases, malaria-visceral leishmaniasis co-infections occur in large populations and exist in different areas even if they have been poorly investigated. The aim of this study was to determine malaria-visceral leishmaniasis co-infection and their associated factors among migrant laborers.

**Methods:**

Community based cross-sectional study was conducted from October–December 2016 on migrant laborers who are residents of rural agricultural camp in West Armachiho district and involved in sesame and sorghum harvesting. Standardized questionnaire was used to collect socio-demographic data and risk factors. Capillary blood was collected for giemsa stained blood film examination to detect and identify *Plasmodium* parasites. Recombinant kinensin (rk39) antigen test was performed to detect anti-leishmania donovani antibody. Data was coded, entered, checked for completeness and analyzed using SPSS version-20 statistical software. Chi-square test was applied to show a significant association between variables. *P*-value < 0.05 was considered as statistically significant.

**Results:**

A total of 178 migrant laborers were included in this study. Of these, 74.2% belong to the age group 15–29; 61.2% come from lowland areas and 51.6% visit the area more than four times. Seroprevalence of visceral leishmaniasis was 9.6% (17/178); and 22.4% (40/178) of tested migrant laborers were found malaria infected. The overall prevalence of malaria-visceral leishmaniasis co-infection was 2.8%. Of the total migrant laborer, 47.8% used bed nets, of them 1.2% were malaria-visceral leishmaniasis co-infected; 72.5% used outdoor sites as usual sleeping site, among them 3.1% were malaria-visceral leishmaniasis co-infected; 60.1% were migrants, of which 2.8% were malaria-visceral leishmaniasis co-infected**.** All variables were not significantly associated with malaria-visceral leishmaniasis co-infection (*P* > 0.05).

**Conclusions:**

Prevalence of malaria-visceral leishmaniasis co-infection was low and it is not significantly associated with residence, number of visits, bed net utilization and outdoor sleeping habit even if both diseases are prevalent in the study area.

## Background

Malaria is an acute or chronic mosquito born tropical disease caused by intracellular protozoan parasites of the genus *Plasmodium* [1]. It is prevalent in tropical and subtropical countries and become a challenge to a highly endemic area of East Africa including Ethiopia [2, 3]. It is transmitted via the bite of infected female anopheles mosquito. Of the 465 properly identified *Anopheles* mosquito species worldwide, 70 have the ability to transmit malaria parasites in humans [4]. In Ethiopia, *Anopheles arabiensis* transmits malaria mainly whereas, *A. pharoensis, A.nili* and *A.funestus* are secondary malaria vectors [5, 6]. *Plasmodium .falciparum, P.vivax*, *P.ovale*, and *P.malariae* are the *Plasmodium* species that infect humans in Africa [7]. *Plasmodium falciparum* is the most prevalent malaria parasite in Sub-Saharan Africa, accounting for 99% of estimated malaria cases in 2016 [1].

According to the 2017 World Health Organization (WHO) malaria report, there were 216 million malaria cases worldwide, accounting for nearly 445,000 deaths, of which 91% were in WHO African region, 6% were in WHO South-East Asian region and 3% were in WHO Eastern Mediterranean region [1]. Malaria is a major public health concern in Ethiopia since it is one of the leading causes of morbidity and mortality especially in migrant laborers, under five children and pregnant women [8, 8].

Leishmaniasis is a neglected tropical vector-borne parasitic disease caused by the intracellular protozoan parasites of the genus *Leishmania* [9, 10]. More than 20 species of the genus *Leishmania* are known to cause leishmaniasis [10]. It is transmitted by the bite of infected female sand flies of the genus *Phlebotomus* or *Lutzomyia* in the old world and new world, respectively [11]. Visceral leishmaniasis or kala-azar is a systemic disease affecting liver, spleen, bone marrow and lymph nodes and caused by *Leishmania donovani* complex with high incidence in East Africa [9, 10, 12]. It is the fatal form of leishmaniasis if left untreated [13].

Visceral leishmaniasis causes an estimated annual incidence of 202,000–400,000 clinical cases and about 20,000–40,000 deaths per year worldwide [10]. Eastern Africa is the second in VL cases, next to Indian sub-continent, with the highest incidence in Ethiopia and Sudan (North and South) [14]. In Ethiopia, it is estimated that each year more than 4000 individuals suffer from visceral leishmaniasis [15]. Highly affected area is Northwest Ethiopia adjoining with Sudan which accounts for more than 60% of the reported visceral leishmaniasis cases [16].

Extensive agricultural farming in Northwest Ethiopia draws over five hundred thousand daily laborers for harvesting of cash crops such as sorghum, sesame and cotton [17]. They usually perform agricultural activities at night time (the time at which vectors are most active) and sleep outdoor where appropriate vector control tools are absent which expose them to sand fly and exophilic-exophagic anopheles mosquito bite [17, 18].

There is geographical overlap between malaria and visceral leishmaniasis in East Africa including Ethiopia and their distribution is greatly influenced by environmental and behavioral factors in addition to distribution of biological insect vector [19]. Malaria-visceral leishmaniasis co-infections are common in East African countries where malaria and visceral leishmaniasis are co-endemic. Their prevalence ranges from 3.8 to 60.8% in Sudan [20] and 19% in Uganda [21]. In Ethiopia, both malaria and visceral leishmaniasis are endemic. However, malaria-visceral leishmaniasis co-infection prevalence remains unown except a study conducted in Metema hospital which showed malaria-visceral leishmaniasis co-infection prevalence of 4.2% [22]. Malaria-visceral leishmaniasis co-Infection impose high impact on public health in general and migrant laborers in particular causing significant death and illness. Beside this, it decrease their working capacity which result in decline of agricultural output and economic crisis to unexpected level.

Despite the current interventions taken to control malaria and leishmaniasis in Ethiopia, it is impossible to eliminate them due to vectors resistance to insecticides, low coverage of preventive tools, large population movement and low access to health care service. The aim of this study was to determine malaria-visceral leishmaniasis co-infection and associated factors among migrant laborers.

## Methods

This study uses methodology from previously published article (Aschale et al) especially in sampling technique, sociodemographic data collection, *Plasmodium* parasite detection, quality control and data analysis.

### Study design

Community based cross-sectional study was conducted among migrant laborers aged 15–65 and engaged in sesame and sorghum harvesting to determine the prevalence of malaria-visceral leishmaniasis co-infection.

### Study setting

The study was conducted in agricultural camps of West Armachiho district, Northwest Ethiopia from October to December 2016. West Armachiho is bordered on the South by Metema, on the West by Sudan, on the North by Tigray region, on the Northeast by Tegede and on the East by Tach Armachiho. According to 2007 national census West Armachiho District has a total population of 31, 730. It has an altitude of 667 m above sea level and minimum annual temperature of 22 °C to 28 °C. The West Armachiho district is one of the areas where large-scale agricultural farming is undertaken.

### Inclusion criteria

Migrant laborers who reside in rural agricultural camps of the district and 15 or more years old were included.

### Exclusion criteria

Migrant laborers who have taken medication for malaria and/or visceral leishmaniasis for the last two weeks were excluded from the study.

### Sample size determination

The required sample size was calculated using a single population proportion statistical formula [n = *z*^2^*p* (1 − p)/d^2^] considering 95% CI and taking prevalence (P) 0.12 from previous study conducted in the same study area [18] and 10% non-response rate.

### Sampling techniques

Proportionate two stage cluster sampling method was used to select study participants. First farm sites were grouped by the name of the owner of farm site. Thirty (30) farm sites were enrolled in the study and among them 11 farm sites were selected by simple random sampling technique using lottery method. One hundred seventy eight (178) study subjects were selected from 11 farm sites proportionally.

### Data collection and laboratory methods

#### Socio-demographic data collection

Information on demographic and risk factors was collected using Amharic version standardized questionnaire. Required information was gathered from labour and social affairs office, health offices, and environmental protection, land administration and use authority at woreda and zonal level. Before the actual data collection, site visit to the enrolled farm sites and discussion with managers was held. Finally, interview was conducted by trained health workers and field research assistants at selected farm sites.

#### *Plasmodium* parasite detection and identification

After interview, blood film examination was performed based on the standard and well accepted guideline [23]. Capillary blood samples were collected from study participants at selected farm sites. Thin and thick blood films were made on a single slide; each slide was labeled, air dried, the thin films were fixed by methanol, then stained with 10% giemsa and examined microscopically using 100 times objective.

#### Detection of anti-leishmania donovani antibody

A single finger prick peripheral blood specimen (8-12 μL) was collected from each study participants and tested by immuno-chromatographic technique (ICT) using rk39 antigen test kit (IT LEISH, France), a 39 amino acid repeat more suitable for field use, having high sensitivity and specificity. Finally the result was interpreted accordingly. To avoid reader variability, it is checked by three senior laboratory professionals.

#### Quality control

Giemsa stock solution was filtered during preparation of working solution, the quality of each batch of working giemsa stain solution was then checked by using known negative and positive blood smear before use. Stained slides were re-checked by two experienced laboratory technologists blindly. To maintain the quality of rk39 test kit, appropriate storage condition was met. Generated data were checked for completeness and cleanness before starting the actual analysis.

### Data analysis and interpretation

Data were entered and analyzed using SPSS version 20.0 (SPSS Inc., Chicago, 2011) software. Then, the study findings were explained in words, tables and graphs. Chi-square test was used for statistical analysis to show presence of significant association between variables. *P*-value < 0.05 was considered as statistically significant.

## Result

### Socio-demographic characteristics

A total of 178 (163 male and 15 female) migrant laborers selected from 11 farm sites were included in this study. Their mean age was 26.1 (range 15–65 years) with standard deviation of 8.6. Among these, 91.6% were male and 74.2% were in the age range of 15–29. Of them, 92.7% were from Amhara region and 60.1% were migrants. About 36.5% of study participants have completed elementary school and 51.6% of migrant laborers visit the area more than four times (Table 1).

**Table 1:** Socio-demographic characteristics of tested migrant laborers in West Armachiho districts, Northwest Ethiopia, 2016 (*N* = 178)

Variables	Category	Frequency	Percentage
Sex	Male	163	91.6
	female	15	8.4
Age group	15–29	132	74.2
	30–44	38	21.3
	45–59	4	2.2
	> = 60	4	2.2
Marital status	Married	47	26.4
	Single	127	71.3
	Widowed	4	2.2
Religion	Orthodox	176	98.9
	Muslim	2	1.1
Educational level	Unable to read and write	13	7.3
	Read and write	58	32.6
	Elementary school	65	36.5
	High school & above	42	23.6
Ethnicity	Tigre	13	7.3
	Amhara	165	92.7
Home area	Highland	69	38.8
	Lowland	109	61.2
Residence	Resident	71	39.9
	Migrant	107	60.1
Visit to the area	First	42	23.6
	Second	17	9.6
	Third	27	15.2
	Fourth or more	92	51.6

### Prevalence of malaria, visceral leishmaniasis and malaria-visceral leishmaniasis coinfection

Of the total tested migrant laborers, 22.4% (*n* = 40), 9.6% (*n* = 17), 2.8% (*n* = 5) were infected with malaria, visceral leishmaniasis, and both malaria and visceral leishmaniasis respectively (Table 2).

**Table 2:** Prevalence of Malaria, Visceral leishmaniasis and Malaria-Visceral leishmaniasis coinfection among migrant laborers in West Armachiho District, Northwest Ethiopia; 2016 (*N* = 178)

Cases	Frequency (n)	Percentage (%)
Malaria		
Positive	40	22.4
Negative	138	77.6
Total	178	100
Visceral Leishmaniasis		
Positive	17	9.6
Negative	161	90.4
Total	178	100
Malaria-VL co-infection		
Yes	5	2.8
No	173	97.2
Total	178	100

*VL Visceral Leishmaniasis

### Plasmodium species proportion

**Fig. 1:**
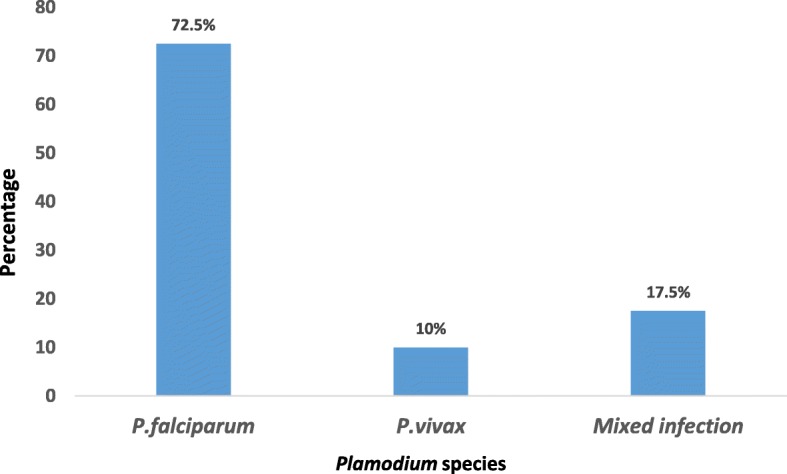
Proportion of *plasmodium* species among positive case (*N* = 40)

Among all positive malaria cases (*n* = 40)**,** *Plasmodium falciparum, Plasmodium vivax* and mixed infection accounts 72.5% (*n* = 29), 10% (*n* = 4) and 17.5% (*n* = 7) respectively (Fig. 1).

### Malaria-visceral leishmaniasis co-infection risk factor analysis

The age group 15–29 comprises 90% of the total positive malaria cases and 80% of malaria- visceral leishmaniasis co-infected migrant laborers. There is significant association between age group and malaria (*P* < 0.05) but there was no significant association between malaria-visceral leishmaniasis co-infection and age group (Table 3).

**Table 3:** Factors associated with malaria-visceral leishmaniasis co-infection among migrant laborers in West Armachiho district, Northwest Ethiopia, 2016 (*n* = 178)

Variables	Category	Malaria-Visceral leishmaniasis co-infection		Chi-square	*P value*
		Yes (%)	No (%)		
Sex	Male	5 (3.1)	158 (96.9)	0.89	0.34
	Female	0 (0)	15 (100)		
Age group	15–29	4 (3)	128 (97)	0.48	0.92
	30–44	1 (2.6)	37 (97.4)		
	45–59	0 (0)	4 (100)		
	> = 60	0 (0)	4 (100)		
Residence	Resident	2 (2.8)	69 (97.2)	0.00	0.99
	Migrant	3 (2.8)	104 (97.2)		
Educational level	Unable to read & write	0 (0)	13 (100)	6.56	0.08
	Read &write	2 (3.4)	56 (96.6)		
	Elementary school	0 (0)	65 (100)		
	High school & above	3 (7.1)	39 (92.9)		
Home area	Highland	1 (1.4)	68 (98.6)	0.83	0.36
	Lowland	4 (3.7)	105 (96.3)		
Use bed net	Yes	1 (1.2)	84 (98.8)	1.71	0.19
	No	4 (4.3)	89 (95.7)		
Usual sleeping accommodation	Outdoor	4 (3.1)	125 (96.9)	0.15	0.69
	Indoor	1 (2)	48 (98)		
Visit to the area	First	0 (0)	42 (100)	7.74	0.10
	Second	0 (0)	17 (100)		
	Third	3 (11.1)	24 (88.9)		
	Forth or more	2 (2.2)	90 (97.8)		

From the total study participants, 85 (47.8%) used bed nets. Of these, 1 (1.2%) migrant laborer were malaria-visceral leishmaniasis co-infected. Majority of the migrant laborers; 129 (72.5%) used outdoor sites as usual sleeping accommodation, of which 4 (3.1%) were malaria-visceral leishmaniasis co-infected. Among the tested migrant laborers, 60.1% (*n* = 107) were migrants, of which 3 (2.8%) were malaria-visceral leishmaniasis co-infected. All variables were not significantly associated with malaria-visceral leishmaniasis co-infection (*P* > 0.05) (Table 3).

## Discussion

Both malaria and visceral leishmaniasis are endemic in semi-arid and arid areas of the world and are major public health problems causing significant death and illness. They have economic, social and physical impact and impose high burden in developing countries including Ethiopia [24]. Daily laborers visit West Armachiho district, Northwest Ethiopia seeking temporary employment on large scale farms. They usually spend night time in outdoor sites and perform their activities there. Beside this, they have little knowledge about *Anopheles* mosquito and sand fly vectors, and methods of protection from bite. This increases the risk of acquiring both infections [17, 18, 25].

In this study the overall prevalence of malaria-visceral leishmaniasis co-infection was 2.8%. This is comparable with a study conducted among patients attending Metema Hospital; Northwest Ethiopia which is 4.2% [22] and India 5.9% [26] but lower than a study conducted in Uganda 19% [21]. The reason for this discrepancy might be due to difference in endemicity and prevalence of visceral leishmaniasis and malaria between them.

The prevalence of malaria-visceral leishmaniasis co-infection was insignificantly higher in males than females and in the age group 15–29 than other age groups. This is in line with a previous study conducted among patients attending Metema Hospital, Northwest Ethiopia [22]. The reason behind might be due to difference in job distribution and outdoor activities between male and female. Younger male migrant laborers usually perform outdoor activities to harvest and cultivate cash crops exposing themselves for mosquito and sand fly bite whereas female migrant laborers are usually cookers and stay at home. This might reduce the risk of acquiring both infections in females. Malaria-visceral leishmaniasis co-infection was insignificantly higher in migrants than residents. This might be due to the fact that migrants haven’t knowhow in protecting themselves from sand fly and mosquitoes bite and using prevention tools to avoid infection. Since they usually come from malaria and visceral leishmaniasis free highland areas, even they don’t know the burden and endemicity of diseases in the area and fail to apply different prevention tools. Malaria-visceral leishmaniasis co-infection was insignificantly higher in those who use outdoor sites as usual sleeping accommodation than who slept in indoor sites. The reason behind is that; migrant laborers who sleep outdoor are exposed for exophilic-exophagic anopheles mosquito vectors and acquire *Plasmodium* infection easily.

In the present study the prevalence of visceral leishmaniasis was 9.6%. This is consistent with a study conducted in Kafta-Humera low lands; the same geography with study area, which is 12.5% [27] and lower than a study conducted in Metema Hospital with overall prevalence of 22.6% [28]. The reason for this difference might be due to difference in study population, study design and study period.

In this study the prevalence of malaria was 22.4% which is higher than a study conducted among migrant laborers in two districts of North Gondar Zone (Metema and West Armachiho) which is 12% [18] and among patients attending Metema Hospital which is 17% [29]. The reason for this discrepancy might be due to difference in study period, study population, study design and diagnostic tools used. *Plasmodium falciparum* is the most dominant species in the study area which is similar with previous studies conducted in two districts of North Gondar Zone (Metema and West Armachiho) and Metema Hospital [18, 29].

### Strength and limitation of the study

The study was conducted in a very remote area (hard to reach population) where no prevention measure is applied to reduce the burden of both malaria and visceral leishmaniasis in such high risk group of population; and our finding may alert stakeholders to design timely and appropriate intervention strategy. However, we are unable to use molecular technique for parasite detection; and to calculate malaria parasitaemia.

## Conclusion

The prevalence of malaria-visceral leishmaniasis co-infection was low even if the area is endemic for both diseases and it is not associated with residence, number of visits, bed net utilization and outdoor sleeping habit of migrant laborers. Regular health education programs should be organized and implemented for such high risk population to create awareness.

## Abbreviations

VL: Visceral Leishmaniasis
WHO: World Health Organization

## References

[1] World Health Organization (2017). World malaria report 2017.

[2] Martens P, Hall L (2000). Malaria on the move: human population movement and malaria transmission. Emerg Infect Dis.

[3] Krogstad DJ (1996). Malaria as a re-emerging disease. Epidemiol Rev.

[4] Service M (2002). The Anopheles vector in essential malariology.

[5] Federal Democratic Republic of Ethiopia Ministry of Health (2012). National malaria Guidelines.

[6] Federal Democratic Republic of Ethiopia (2015). National strategic plan for malaria prevention, control and elimination in Ethiopia, 2011-2015.

[7] World Health Organization (2003). Malaria entomology and vector control (Learner’s guide).

[8] Rowe AK, Rowe SY, Snow RW, Korenromp EL, Schellenberg JR, Stein C (2006). The burden of malaria mortality among African children in the year 2000. Int J Epidemiol.

[9] World Health Organization (2010). Control of the leishmaniasis: report of a meeting of the WHO expert committee on the control of Leishmaniasis.

[10] Alvar J, Velez I, Bern C, Herrero M, Desjeux P, Cano J (2012). World Health Organization Leishmaniasis control team. Leishmaniasis worldwide and global estimates of its incidence. PLoS One.

[11] Elnaiem DE, Hassan HK, Osman OF, Maingon RD, Killick-Kendrick R, Ward RD (2011). A possible role for Phlebotomus in transmission of *Leishmania donovani*. Parasitol Vectors.

[12] Ritmeijer K, Dejenie A, Assefa Y, Hundie T, Mesure J, Boots G (2006). A comparison of miltefosine and sodium stibogluconate for treatment of visceral leishmaniasis in an Ethiopian population with high prevalence of HIV infection. Clin Infect Dis.

[13] Chappuis F, Sundar S, Hailu A, Ghalib H, Rijal S, Peeling R (2007). Visceral leishmaniasis: what are the needs for diagnosis, treatment and control?. Nat Rev Microbiol.

[14] Hailu A, Balkew M, Berhe N, Meredith SE, Gemetchu T (1995). Is *Phlebotomus (Larroussius) orientalis* a vector of visceral leishmaniasis in south-West Ethiopia?. Acta Trop.

[15] Federal Democratic Republic of Ethiopia Ministry of Health (2006). National guidelines for diagnosis and treatment of leishmaniasis.

[16] Mengistu G, Ayele B (2007). Visceral leishmaniasis and HIV co-infection in patients admitted to Gondar University Hospital, Northwest Ethiopia. Ethiop J health develop.

[17] Adhanom T, Deressa W, Witten H, Getachew A, Seboxa T, Birhanie Y, Hailemariam D, Kloos H (2006). Malaria: In the Epidemiology and Ecology of Health and Disease in Ethiopia.

[18] Schicker R, Hiruy N, Melak B, Gelaye W, Bezabih B, Stephenson R (2015). A venue-based survey of malaria, anemia and mobility patterns among migrant farm workers in Amhara region, Ethiopia. PLoS One.

[19] Gebre-Michael T, Malone B, Balkew M, Ali A, Berhe N, Hailu A (2004). Mapping the potential distribution of *Phlebotomus martini* and *Phlebotomus orientalis* (Diptera: Psychodidae), vectors of kala-azar in East Africa by use of geographic information systems. Acta Trop.

[20] Van den Bogaart E, Berkhout MM, Nour AB, Mens PF, Talha A-BA, Adams ER (2013). Concomitant malaria among visceral leishmaniasis in-patients from Gedarif and Sennar states, Sudan: a retrospective case-control study. BMC Public Health.

[21] Van den Bogaart E, Berkhout MM, Adams ER, Mens PF, Sentongo E, Mbulamberi DB (2012). Prevalence, features and risk factors for malaria co-infections amongst visceral leishmaniasis patients from Amudat Hospital, Uganda. PLoS Negl Trop Dis.

[22] Ferede G, Diro E, Getie S, Getnet G, Takele Y, Amsalu A, et al. Visceral Leishmaniasis-malaria co-infection and their associated factors in patients attending Metema Hospital, Northwest Ethiopia. Suggestion for integrated vector management. Malar Res Treat. 2017;2017. 10.1155/2017/6816913 PMC5592390 28932617

[23] Cheesbrough M. District laboratory practice in tropical countries: Cambridge university press; 2006.

[24] Consortium M. Leishmaniasis control in eastern Africa: Past and present efforts and future needs. Situation and gap analysis. 2010;86.

[25] Argaw D, Mulugeta A, Herrero M, Nombela N, Teklu T, Tefera T (2013). Risk factors for visceral leishmaniasis among residents and migrants in Kafta-Humera, Ethiopia. PLoS Negl Trop Dis.

[26] Nandy A, Addy M, Guha S, Maji A, Chauahuri D, Chatterjee P (1995). Co-existent kala-azar and malaria in India. Trans R Soc Trop Med Hyg.

[27] Lemma W, Tekie H, Yared S, Balkew M, Gebre-Michael T, Warburg A (2015). Sero-prevalence of *Leishmania donovani* infection in labour migrants and entomological risk factors in extra-domestic habitats of Kafta-Humera lowlands-kala-azar endemic areas in the Northwest Ethiopia. BMC Infect Dis.

[28] Shiferaw Y, Wondimeneh Y, Wondifraw H, Ferede G. Trend analysis of visceral Leishmaniasis in Metema Hospital, Northwest Ethiopia. J Epidemiol Public Health Rev. 2016;1(5). 10.16966/2471-8211.129.

[29] Ferede G, Worku A, Getaneh A, Ahmed A, Haile T, Abdu Y, et al. Prevalence of malaria from blood smears examination: a seven-year retrospective study from Metema Hospital, Northwest Ethiopia. Malar Res Treat. 2013;2013. 10.1155/2013/704730 PMC3876907 24455415

